# Effects of Trigger Point Dry Needling for the Management of Knee Pain Syndromes: A Systematic Review and Meta-Analysis

**DOI:** 10.3390/jcm9072044

**Published:** 2020-06-29

**Authors:** Youssef Rahou-El-Bachiri, Marcos J. Navarro-Santana, Guido F Gómez-Chiguano, Joshua A Cleland, Ibai López-de-Uralde-Villanueva, César Fernández-de-las-Peñas, Ricardo Ortega-Santiago, Gustavo Plaza-Manzano

**Affiliations:** 1 Escuela Internacional de Doctorado, Universidad Rey Juan Carlos (URJC), Alcorcón, 28922 Madrid, Spain; baray_09@hotmail.com; 2 Department of Radiology, Rehabilitation and Physiotherapy, Faculty of Medicine, Universidad Complutense de Madrid, 28040 Madrid, Spain; marcosjose.navarrosantana@gmail.com (M.J.N.-S.); ibailope@ucm.es (I.L.-d.-U.-V.); gusplaza@ucm.es (G.P.-M.); 3 Rehabilitación San Fernando, 28807 Madrid, Spain; 4 Clínica Dinamia Fisioterapia, 28806 Madrid, Spain; guido.gomez.ft@gmail.com; 5 Doctor of Physical Therapy Program, Department of Public Health and Community Medicine, Tufts University School of Medicine, Boston, MA 02111, USA; joshcleland@comcast.net; 6 Department of Physical Therapy, Occupational Therapy, Physical Medicine and Rehabilitation, Faculty of Health Sciences, Universidad Rey Juan Carlos (URJC), Alcorcón, 28922 Madrid, Spain; ricardo.ortega@urjc.es; 7 Cátedra Institucional en Docencia, Clínica e Investigación en Fisioterapia: Terapia Manual, Punción Seca y Ejercicio Terapéutico, Universidad Rey Juan Carlos, Alcorcón, 28922 Madrid, Spain; 8 Instituto de Investigación Sanitaria del Hospital Clínico San Carlos, 28040 Madrid, Spain

**Keywords:** dry needling, knee pain, musculoskeletal pain, meta-analysis

## Abstract

Background: To evaluate the effect of trigger point dry needling alone or as an adjunct with other interventions on pain and related disability in people with knee pain. Methods: Several electronic databases were searched for randomized controlled trials where at least one group received dry needling for knee pain. Studies had to include human subjects and collect outcomes on pain and pain-related disability in musculoskeletal knee pain. Data were extracted by two reviewers. The risk of bias was assessed by the Cochrane Guidelines, methodological quality was assessed with the Physiotherapy Evidence Database (PEDro) score, and the quality of evidence by using the GRADE approach. Standardized mean differences (SMD) were calculated. Results: Ten studies (six patellofemoral pain, two knee osteoarthritis, two post-surgery knee pain) were included. The meta-analysis found moderate effect sizes of dry needling for reducing pain (SMD −0.53, 95% CI −0.87 to −0.19) and improving related disability (SMD −0.58, 95% CI −1.08 to −0.09) as compared to a comparison group at short-term. The main effect was observed for patellofemoral pain (SMD −0.64, 95% CI −1.17 to −0.11). No significant effects were observed at mid- or long-term follow-ups. The risk of bias was generally low, but the heterogenicity and the imprecision of the results downgraded the level of evidence. Conclusion: Low to moderate evidence suggests a positive effect of trigger point dry needling on pain and related disability in patellofemoral pain, but not knee osteoarthritis or post-surgery knee pain, at short-term. More high-quality trials investigating long-term effects are clearly needed.

## 1. Introduction

Knee pain is a symptom accounting for approximately one third of musculoskeletal presentations seen in primary care. [1] Within the Johnston County Osteoarthritis (JoCo OA) Project, the overall prevalence of knee pain was 43.3% [2]. Patellofemoral pain (PFP) and knee osteoarthritis (OA) are probably the most common causes of knee pain symptoms of musculoskeletal origin. A recent meta-analysis reported an annual prevalence for PFP of 22.7% in the general adult population and of 28.9% in adolescents [3]. It appears that PFP exhibits a female preponderance with a female: male ratio of 2.2:1 [4]. Similarly, knee OA was ranked as the 11th highest contributor to global disability in the Global Burden of Disease Study [5]. In fact, in the JoCo OA Project, the overall prevalence of knee OA was 27.8%, again, with a higher prevalence in women (31.0%) [2]. The prevalence of knee OA can reach up to 30% in former athletes [6].

Conservative treatment is the first therapeutic option for the management of people with PFP or knee OA; however, the most appropriate treatment strategy remains unclear. In fact, different interventions including injections, medication, exercise, manual therapy, physical activity, education, and tape are recommended, but their levels of evidence are heterogeneous [7,8]. There is evidence supporting a role of the quadriceps musculature in both PFP and knee OA. In fact, quadriceps weakness has been found to be a potential risk factor for development of PFP in adolescents [9] and development of knee OA in adults [10]. In such a scenario, it has been proposed that myofascial trigger points (TrPs) could contribute to sensory and motor disturbances observed in knee pain disorders [11,12]. A TrP is defined as a hypersensitive spot in a taut band of a skeletal muscle, in which stimulation can induce sensory and motor disturbances [12]. Sensory symptoms associated with TrPs include the presence of spreading referred pain and hyperalgesia [12], whereas motor disturbances include accelerated muscle fatigability [13] or increased antagonist co-activation [14]. Preliminary evidence has found that the referred pain elicited by TrPs in the knee and hip muscles can contribute to PFP [15] and knee OA [16] symptoms. 

Several therapeutic approaches are proposed for the management of TrPs, with dry needling receiving an increased interest in the literature [17]. Dry needling is a “skilled intervention using a thin filiform needle to penetrate the skin that stimulates myofascial TrPs, muscles, and connective tissue for the management of musculoskeletal disorders” [18]. The aim of dry needling is to inactivate the altered muscle dysfunction induced by the presence of a TrP and to improve function [17]. It is important to differentiate between wet needling, procedures including the injection of a substance into a TrP area through a hypodermic beveled, cutting edge needle, and dry needling, an intervention including the insertion of a solid filiform needle (usually an acupuncture needle) into the TrP area without the introduction of any substance [17]. Although there is some evidence suggesting a potential positive, but small, effect of dry needling for the treatment of musculoskeletal pain in the lower extremity [19,20], no meta-analysis has specifically investigated the effects of dry needling for the treatment of knee pain conditions. The Consensus published by Collins et al [21] concluded that there is uncertainty regarding the use of needling therapies for the management of patients with PFP; however, this review mixed acupuncture and dry needling strategies. Although acupuncture and dry needling are therapeutic approaches using similar needles, several differences including stimulation points (acupuncture point vs. TrP areas), clinical reasoning framework (Traditional Chinese Medicine vs. Pain Neurosciences Reasoning), needling procedure (twisting vs. peppering) or time retention (20–30 minutes vs. no retention), can be observed [17]. 

Therefore, this systematic review and meta-analysis evaluates the effects of TrP dry needling alone or as an adjunct with other interventions on pain intensity and pain-related disability in individuals with knee pain.

## 2. Experimental Section

This systematic review and metanalysis adheres to the Preferred Reporting Items for Systematic Reviews and Meta-Analyses (PRISMA) statement [22]. The international Open Science Framework Registry link is https://doi.org/10.17605/OSF.IO/3FDVN. 

### 2.1. Systematic Literature Search 

Electronic literature searches were conducted on MEDLINE, CINAHL, PubMed, PEDro, Cochrane Library, SCOPUS and Web of Science databases from their inception to the 20th of April 2020. When searched databases allowed limits, searches were restricted to randomized clinical trials. We also screened the reference lists of the papers that were identified in database searches. Bibliographical database search strategies were conducted with the assistance of an experienced health science librarian.

Population: Adults with knee pain of musculoskeletal origin older than 18 years of age. For this aim, the search strategy had to include one of the following key words: knee pain OR patellofemoral pain OR knee osteoarthritis OR knee arthroplasty OR knee tendinopathy OR knee ligament injuries OR knee meniscal injuries.

Intervention: Any form of muscular (or tendon) dry needling. Acupuncture was excluded. For this aim, the search strategy had to include: dry needling OR muscular needling OR intramuscular stimulation.

Comparator: Acceptable comparators were any type of placebo, sham, or no intervention. For this aim, the search strategy included one of these key words: sham OR placebo OR control OR no intervention. We also included a comparison of dry needling with another intervention.

Outcomes: The primary outcome measure was pain OR related disability.

The search strategy for each database is available in Table 1.

### 2.2. Selection Criteria

The systematic review included randomized clinical trials that recruited people with a chronic knee pain condition of musculoskeletal origin and where at least one group received any form of dry needling. Due to the heterogeneity in the terminology, we included the following diagnosis: knee pain, patellofemoral pain, knee osteoarthritis, knee arthroplasty, knee tendinopathies, ligament injuries, and knee meniscal injuries.

The specific inclusion criteria included 1, adult population (>18 years old) with knee pain of musculoskeletal origin; 2, one group receiving any type of muscle/tendon dry needling intervention; 3, acceptable comparator with sham, placebo, control, or other active intervention; and 4, the primary outcome of the study should include pain intensity (e.g., as measured with a visual analogue scale or a numerical pain rate scale) or related disability (e.g., as assessed with a specific-disease questionnaire). We excluded clinical trials including: (1) knee pain related to neurological disorders (e.g., post-stroke pain); (2) knee pain of non-musculoskeletal origin (e.g., cancer or visceral disorders); (3) studies not published as a journal article; (4) retrospective designs; (5) pilot studies, (6) needling using a Traditional Chinese Medicine Approach, i.e., acupuncture; or (7) use of other injection therapy, e.g., corticoid injection or platelet rich plasma, in the dry needling group.

### 2.3. Screening, Selection Process and Data Extraction

Articles identified from the different databases were independently reviewed by two authors. Duplicate papers were first removed. Second, titles/abstracts were screened for potential eligibility. Third, full-text reads of potentially eligible studies were conducted. Authors were required to achieve a consensus on the potential included trials. In the case of discrepancy, a third author participated to reach the consensus for including the study or not.

Data from each trial including study design, sample size, population, interventions, outcomes, and follow-ups were extracted independently by 2 authors in a standardized form. Both authors had to achieve a consensus on each item on the data-extraction form. If disagreement occurred, a third author participated in the determination.

### 2.4. Assessment of Methodological Quality and Risk of Bias 

Risk of bias and methodological quality of the included trials were independently assessed by two authors using the Cochrane Risk of Bias (RoB) assessment tool [23] and the Physiotherapy Evidence Database (PEDro) scale [24], respectively.

The RoB tool includes the following items: selection bias (randomization sequence generation, allocation concealment), performance bias (blinding participants, blinding therapists), detection bias (blinding outcome assessor), attrition bias (incomplete outcome data), reporting bias (source of funding bias/selecting outcome reporting), and other bias (sample size) [23]. Each item was classified as low risk, high risk or unclear according to the Cochrane Collaboration’s tool [23].

The PEDro score evaluates the quality of the trial by assessing random allocation; concealed allocation; baseline between-groups similarity; participants blinding; therapists blinding; assessors blinding; dropouts; intention-to-treat statistical analysis; between-groups statistical comparison; point measures and variability data [24]. A trial was considered to be of high-quality when the PEDro score is ≥ 5 over a total of 10 points.

### 2.5. Level of Evidence 

To evaluate the quality of the evidence, we used the Grading of Recommendations Assessment, Development, and Evaluation (GRADE) approach [25]. The level of evidence was classified as high, moderate, low or very low based on the following items: presence of study limitations (RoB), indirectness of evidence, inconsistency of results/unexplained heterogeneity, imprecision of results, and high probability of publication bias [26]. This process was independently performed by two authors, with the participation of a third one if discrepancy occurred.

### 2.6. Data Synthesis and Analysis

The meta-analysis was conducted using the Review Manager statistical software (RevMan version 5.3). Data synthesis was categorized by groups according to the follow-up period as short-, mid-, and long-term, if data were available. 

We extracted the sample size, means and standard deviations for each variable. When the trial reported only standard errors, they were converted to standard deviations. When necessary, the mean scores and standard deviations were estimated from graphs. In addition, if the trial presented non-parametric values (median and interquartile range), they were converted to means and standard deviations accordingly [27,28].

The between-groups mean differences (MD) of the trials were converted to SMD, with their 95% confidence intervals (CI). A random-effects model was used to determine the overall effect size (SMD). An effect size (SMD) of 0.8 or greater was considered large, between 0.5 to 0.8 as moderate and between 0.2 to 0.5 as small. In general, *p*-values < 0.05 were considered statistically significant. The overall effect sizes and calculation of the effect size on pain intensity and pain-related disability were obtained at short- (0–10 weeks), mid- (10–20weeks) and long- (>20 weeks) terms post-intervention. 

The heterogeneity of the studies was assessed using the I^2^ statistic. The Cochrane group has established the following interpretation of the I^2^ statistic: 0%–40% may not be relevant/important heterogeneity; 30%–60% suggests moderate heterogeneity, 50%–90% represents substantial heterogeneity, and 75–100% represents considerable heterogeneity [29]. 

## 3. Results

### 3.1. Study Selection

The electronic searches identified 253 potential studies for review. After removing duplicates, 145 studies remained. One hundred and twenty-two (n = 122) were excluded based on examination of titles or abstracts, leaving 20 articles [30,31,32,33,34,35,36,37,38,39,40,41,42,43,44,45,46,47,48,49] for full-text analysis. 

**Figure 1:**
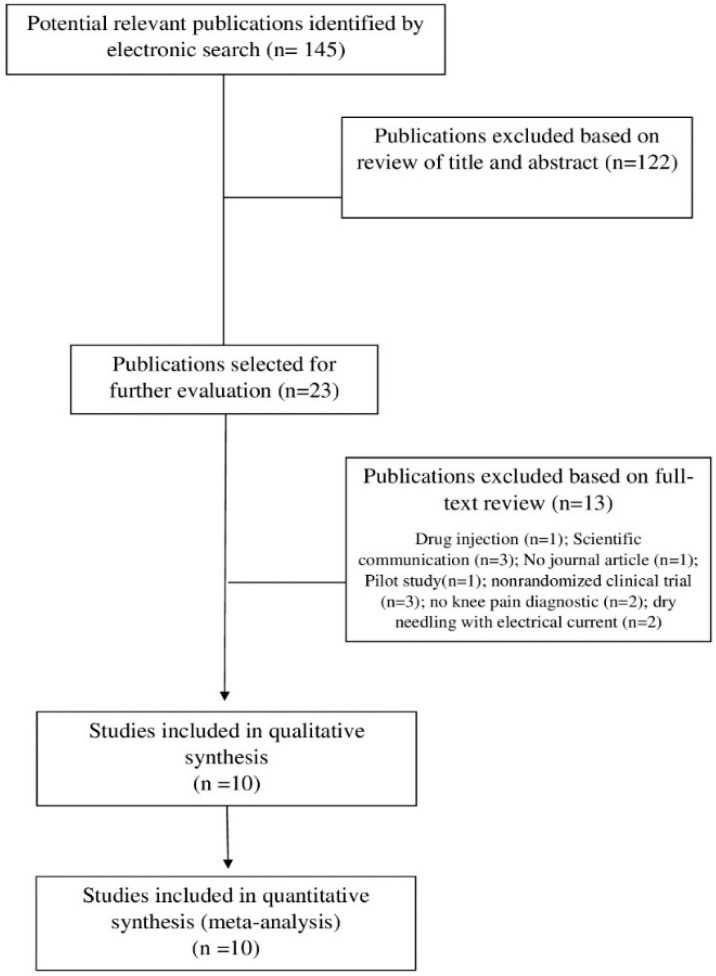
Preferred Reporting Items for Systematic Reviews and Meta-Analyses (PRISMA) Flow diagram.

Ten articles were excluded because the dry needling intervention was combined with another injection therapy [30], abstract/conference proceedings [31,32,33], a pilot study [34], non-randomized clinical trials [35,36,37], and application of dry needling with electrical current [38,39]. Finally, a total of 10 trials [40,41,42,43,44,45,46,47,48,49] were included in the main analyses (Figure 1).

### 3.2. Study Characteristics

The characteristics of the participants of the included studies are shown in Table 2. Six studies (60%) investigated the effects of TrP dry needling in PFP [40,41,43,46,48,49], two (20%) in subjects with knee OA [42,45], and the remaining two (20%) in post-surgery knee pain [44,46].

Most of the studies [41,42,43,44,45,46,47,48,49] targeted active TrPs (i.e., those in which referred pain reproduced the patient’s knee symptoms) with the needle, whereas one trial [40] targeted specific points chosen based on the most common places of TrPs at the quadriceps muscle, as originally described by Simons et al. [12] The needling technique was homogenous; eight trials [41,42,43,44,45,46,47,48] used reported the presence of local twitch responses during the intervention, one [49] did not report it and the last one used superficial, not deep, dry needling [40]. However, there was heterogenicity in the number and frequency of sessions and the type of sham or comparator. Table 3 details the characteristics of the dry needling intervention applied on each trial.

### 3.3. Methodological Quality

The methodological quality scores ranged from 5 to 9 (mean: 7.6, SD: 1.3) out of a maximum of 10 points; therefore, all studies were considered of high methodological quality (≥5 points). The most frequent biases were blinding of the therapists, followed by participant’s blinding. Table 4 represents the details of the PEDro scale of each trial.

### 3.4. Risk of Bias

**Figure 2:**
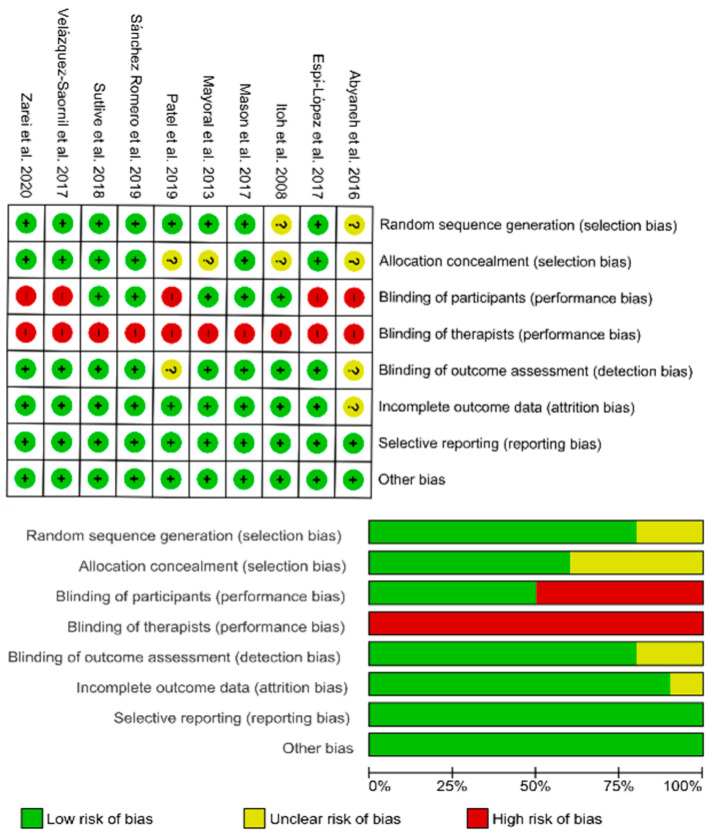
Plots of risk of bias of the included studies.

The risk of bias assessment of the included trials is displayed in Figure 2. No trial was able to blind therapists, five trials had a high bias in the item of blinding participants, and four trials had an unclear bias in the item of allocation concealment. In general, the risk of bias of the included trials in the current meta-analysis was low.

### 3.5. Effects of Dry Needling on Knee Pain Intensity 

**Figure 3:**
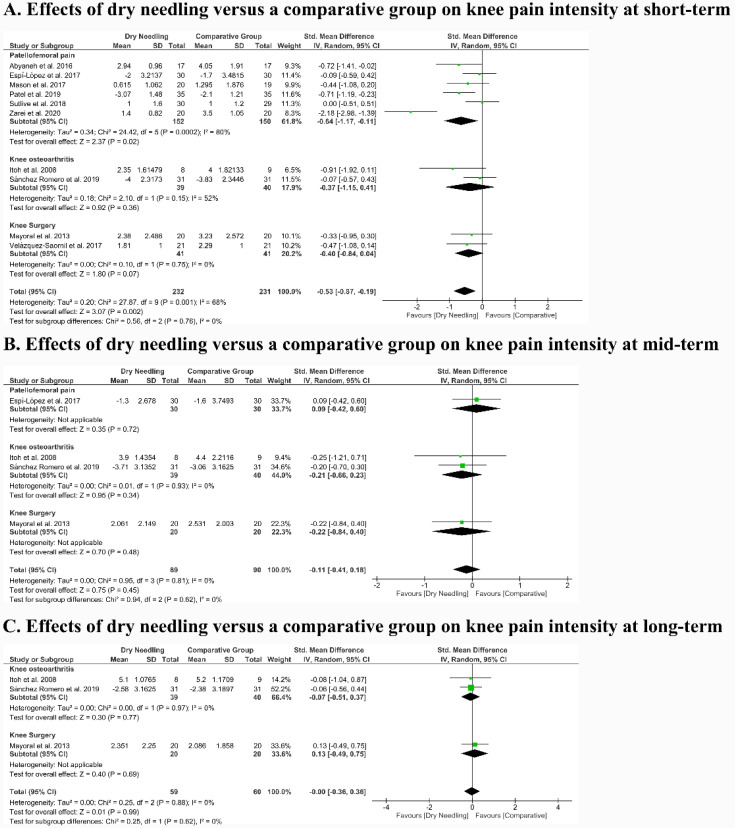
Comparison (standardized mean difference) between the effects of dry needling versus a comparative group on knee pain intensity at (**A**) short- (**B**) mid- and (**C**) long-term. The area of each square is proportional to the study’s weight in the meta-analysis. The horizontal bars represent the confidence intervals of the between-groups difference of the study. The diamond represents the overall meta-analyzed measure of effect (SMD) and the lateral points indicate the confidence intervals for this estimate.

The meta-analysis found that dry needling exhibited a significant moderate effect size (SMD −0.53, 95% CI −0.87 to −0.19, n = 463, Z = 3.07, *p* = 0.002) for decreasing pain intensity versus a comparative group at short-term, but with high heterogeneity (I^2^ = 68%) between studies (Figure 3A). The overall mean difference was −0.85 (95% CI −1.35 to −0.34) points on a 0–10 numerical pain rate scale. No significant differences between subgroups (*p* = 0.76, I^2^ = 0%) were observed. Dry needling showed a significant moderate effect size (SMD −0.64, 95% CI −1.17 to −0.11) with a mean difference of −0.92 (95% CI −1.64 to −0.21) only for PFP. Significant differences between subgroups (*p* = 0.76, I^2^ = 0%) were observed. Dry needling showed a significant moderate effect size (SMD −0.64, 95% CI −1.17 to −0.11) with a mean difference of −0.92 (95% CI −1.64 to −0.21) only for PFP. 

The meta-analysis did not reveal a significant effect of dry needling at mid- (SMD −0.11, 95% CI −0.11 to 0.18, n = 179, Z = 0.75, *p* = 0.45, Figure 3B) and long- (SMD −0.00, 95% CI −0.73 to 0.72, n = 119, Z = 0.01, *p* = 0.99, Figure 3C) terms for decreasing pain intensity versus a comparative group, with null heterogeneity (I^2^ = 0%) between trials. The overall mean difference was -0.35 (95% CI −1.12 to 0.41) and −0.00 (95% CI −0.73 to 0.72) points on a 0–10 numerical pain rate scale at mid- and long-terms, respectively. No differences between subgroups (*p* > 0.05, I^2^ = 0%) were found in both meta-analyses. Table 5 shows the main findings of the included studies.

### 3.6. Effects of Dry Needling on Related Disability

**Figure 4:**
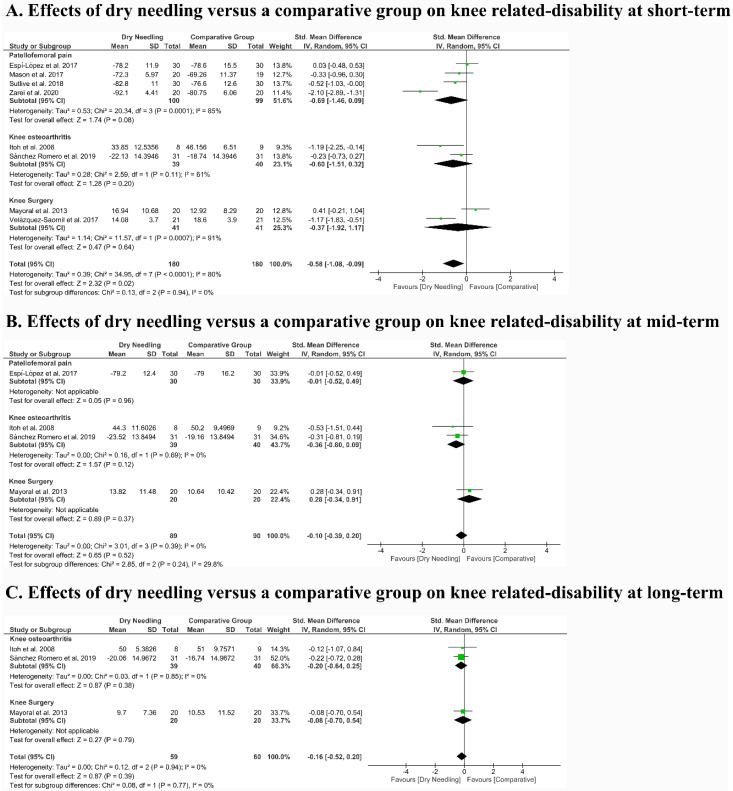
Comparison (standardized mean difference) between the effects of dry needling versus a comparative group on pain-related disability at (**A**) short- (**B**) mid- and (**C**) long-term. The area of each square is proportional to the study’s weight in the meta-analysis. The horizontal bars represent the confidence intervals of the between-groups difference of the study. The diamond represents the overall meta-analyzed measure of effect (SMD) and the lateral points indicate the confidence intervals for this estimate.

The meta-analysis found that dry needling exhibited a significant moderate effect size (SMD −0.58, 95% CI −1.08 to −0.09, n = 360, Z = 2.32, *p* = 0.02) on related disability vs. a comparative group but with high heterogeneity (I^2^ = 80%) between studies (Figure 4A) at short-term. No significant differences between subgroups existed (*p* = 0.94, I^2^ = 0%). Dry needling did not show a significant effect at a mid- (SMD −0.10, 95% CI −0.39 to 0.20, n = 179, Z = 0.65, *p* = 0.52, Figure 4B) and long (SMD −0.16, 95% CI −0.52 to 0.20, n = 119, Z = 0.87, *p* = 0.39, Figure 4C) on pain-related disability vs. comparative group, with null heterogeneity (I^2^ = 0%) between studies. No differences between subgroups (*p* > 0.05, I^2^ < 30%) were found in both meta-analyses. Table 5 shows the main findings of the included studies.

### 3.7. Quality of Evidence (GRADE)

Table 6 displayed the details of GRADE assessment showing RoB, inconsistency of the results, indirectness of evidence, imprecision of results, and high probability of publication bias. 

### 3.8. Adverse Events

Three trials did not provide data on side effects or adverse events [40,42,44]. Espí-López et al [41] reported that 40% of patients experienced post-needling soreness, which resolved spontaneously within 36–48 hours, but no serious adverse events. Velázquez-Saornil et al [47] reported three (13.6%) adverse events (hemorrhages), with one participant withdrawn at follow-up due to this adverse event. Sanchez-Romero et al [45] reported that 87% of the patients experienced minor side effects, 97% being post-needling muscle soreness. Zarei et al. [48] reported a minimal soreness after dry needling intervention but not any serious adverse events. The remaining three studies [43,46,49] reported no adverse events.

## 4. Discussion

### 4.1. Effectiveness of Trigger Point Dry Needling

The objective of this meta-analysis was specifically to investigate the effects of TrP dry needling for the management of knee pain conditions. We found low to moderate evidence suggesting a positive effect of TrP dry needling for pain and related disability, in patients with knee pain. The main effect was observed for patients with PFP, but not in those with knee OA or post-surgery knee pain, at short-term. 

Preliminary evidence has suggested a potential positive effect of dry needling for the treatment of musculoskeletal pain in the lower extremity [19,20]; however, these reviews only included a small number of studies (n = 2) on knee pain. The Consensus published by Collins et al [21] on individuals with PFP did not find evidence supporting the use of needling interventions for this knee pain condition, but these authors combined dry needling with acupuncture. Our meta-analysis is the first specifically analyzing the impact of TrP dry needling on pain intensity and related disability in knee pain of musculoskeletal origin. The results suggest that TrP dry needling may be effective for the management of pain and related disability associated with knee pain (low to moderate evidence); however, most effects were observed at short-term and particularly in PFP, but not in knee OA or post-surgery knee pain. 

Five trials out of six (83%) investigating the effects of dry needling on PFP applied the needing approach combined with other interventions, particularly manual therapy or exercise. It is important to consider that clinicians do not apply just one treatment for pain management and multimodal approaches are preferred. For instance, evidence supporting the use of both hip and knee exercises for managing PFP is high [21,50]. Therefore, it is difficult to determine the isolated effects of TrP dry needling on clinical outcomes in PFP. Since PFP is generally associated with motor [9] and structural [51] disturbances in the surrounding knee and hip muscles, it is probable that the application of dry needling should also be complemented with exercise programs. Most studies including patients with PFP used the TrP hypothesis for needling application and the intervention was applied by a physical therapist [19]. However, muscles receiving the needling intervention were heterogeneous and included hip (e.g., gluteus medium, iliopsoas) and/or knee (e.g., vastus medialis, hamstrings, adductors) muscles. It would be helpful to determine which muscles are more relevant for PFP to explore future consistent protocols for the application of dry needling interventions in this population. 

We did not find a significant effect of TrP dry needling for the management of knee OA or post-surgery knee pain. There are several potential explanations for this lack of effect. First, the small number of studies. Only two trials [42,45] investigated the effects of dry needling on knee OA, whereas another two [44,46] analyzed the effects on post-surgery knee pain, one in patients receiving a total knee replacement and the other in patients after anterior cruciate ligament reconstruction. Second, it is important to consider that these knee pain conditions are related to joint damage, which is associated with an arthrogenic inhibition of the surrounding musculature [52,53]. Therefore, it is possible that TrPs can be perpetuated by this arthrogenic inhibition in a vicious cycle and that several other factors can promote their activity. Third, there are also structural changes in the knee muscles associated with knee OA. For instance, a recent meta-analysis observed that individuals with knee OA exhibit intermuscular fat atrophy [54]. Since TrPs are not associated with muscle atrophy, more complex mechanisms can be involved in knee OA and post-surgical knee pain. Most trials (n=3, 75%) applied the needling intervention to the knee musculature; a recent meta-analysis reported that the inclusion of exercises targeting the hip musculature resulted in greater improvements in pain and function in patients with knee OA [55]. Another explanation may be that knee OA or post-surgery knee pain can also have a neuropathic mechanism (up to 25% of the patients) [56], not influenced by the effects of dry needling. Therefore, the complex underlying mechanisms associated with knee OA pain and post-surgical knee pain could explain the lack of effects of just TrP dry needling. 

### 4.2. Safety of Trigger Point Dry Needling

Since dry needling is an invasive intervention, it is important to consider its safety. Most studies did not report the presence of any adverse event different than post-needling soreness. A recent study investigating adverse events of dry needling reported that most adverse events are categorized as minor with the top three adverse events being bleeding (16%), bruising (7.7%), and pain during the intervention (5.9%) [57]. Nevertheless, some major adverse events can also occur, depending on the anatomical location. Some case reports have documented the presence of infection after application of dry needling [58,59]. These are uncommon complications of dry needling; therefore, sterilization of the dry needling targeted area is important to minimize the risk of infection and assure proper safety of the technique. Although dry needling seems to be a safe intervention if properly applied, therapists need to be aware of the potential risks associated with its application on each body area where it is applied. 

### 4.3. Strengths and Limitations

Although this is the first meta-analysis specifically analyzing the effects of TrP dry needling in patients with knee pain of musculoskeletal origin, the current results should be generalized within the context of its strengths and limitations. The sstrengths of the current meta-analysis include a comprehensive literature search, methodological rigor, data extraction, rigorous statistical analysis, and the inclusion of only randomized controlled trials of high methodological quality. In fact, the current systematic review and meta-analysis (level 1a evidence) should be integrated into the evidence-based medicine (EBM) framework since it integrates data from randomized controlled trials (level 1b evidence) by also using grading recommendations for its conclusions. 

Among the limitations, the number of the included trials for knee OA or post-surgical pain was small (n = 2). Additionally, dry needling interventions were applied with different dosages, i.e., sessions and frequency of application, and in different muscles, explaining the heterogeneity and imprecision of the results of some of the trials. It would be interesting to better define the interventions applied for potential replication of the treatment protocols. Therefore, the results of the current meta-analysis should be considered with caution. 

### 4.4. Clinical and Research Implications

The current meta-analysis found low to moderate evidence supporting the use of TrP dry needling for the treatment of PFP, but not for knee OA or post-surgical knee pain; but several questions remain to be elucidated in future studies. First, most studies investigated short-term effects, with only two studies investigating longer follow-ups for each knee pain condition. Therefore, there is a clear need for randomized clinical trials examining long-term effects of dry needling, combined with exercise interventions, for knee pain conditions. 

The topic of a proper sham needling intervention should also be considered, since it is not possible to determine that real dry needling is superior to sham dry needling. In fact, Braithwaite et al concluded that sham needling interventions used in clinical trials are diverse, limiting the comparability of blinding effectiveness across current studies [60].

Finally, other potential knee pain conditions, e.g., patellar tendinopathy, could also benefit from TrP dry needling; however, no clinical trial was included in the current meta-analysis. A systematic review reported that tendon dry needling improved patient-reported outcome measures in individuals with tendinopathy; however, this review only included four studies and none on patellar tendinopathy [61]. The recent review conducted by Mendonça et al found one study showing a potential positive effect of dry needling in patients with patellar tendinopathy [62], but this study was excluded from our analysis because the needling intervention was combined with another injection therapy [30].

## 5. Conclusions

Based on the results of individual randomized controlled trials included and on the overall effect size derived from the current meta-analysis, we recommend (moderate evidence) the application of TrP dry needling as compared to other treatments for short-term reduction of pain in individuals with knee pain of musculoskeletal origin. The meta-analysis revealed that TrP dry needling was effective for decreasing pain in PFP, but not with in knee OA or post-surgical knee pain. 

## Figures and Tables

**Table 1:** Database formulas during literature search.

**PubMed Search Formula**
#1 "Patellofemoral Pain Syndrome"(MeSH Terms) OR "Chondromalacia Patellae"(MeSH Terms) OR "Osteoarthritis, Knee"(MeSH Terms) OR "Knee Osteoarthritis" OR "Arthroplasty, Replacement, Knee"(MeSH Terms) OR "Knee Arthroplasty" OR "Knee Prosthesis"(MeSH Terms) OR "Knee Injuries"(MeSH Terms) OR "Anterior Cruciate Ligament Injuries"(MeSH Terms) OR "ACL Injury" OR "Medial Collateral Ligament Knee Injury" OR "Knee Joint Injury" OR "Knee Dislocation"(MeSH Terms) OR "Meniscectomy"(MeSH Terms) OR "Meniscus Injury" OR "Tibial Meniscus Injuries"(MeSH Terms) OR "Meniscus Tear" OR "Bucket Handle Tear" OR "Flap Tear" OR "Patellar Tendinopathy" OR "Patellar Tendonitis" OR "Patellar Tendinosis" OR "Jumper Knee" #2 "Dry Needling"(Mesh) OR "Intramuscular Stimulation" (Title/Abstract) OR "Muscular Needling" (Title/Abstract) #3 #1 AND #2
Results: 37
**CINAHL/Medline Search Formula (EBSCO)/WOS Search Formula**
("Patellofemoral Pain Syndrome" OR "Chondromalacia Patellae" OR "Knee Osteoarthritis" OR "Knee Arthritis" OR "Knee Arthroplasty" OR "Knee Prosthesis" OR "Knee Injury" OR "Anterior Cruciate Ligament Injury" OR "ACL Injury" OR "Medial Collateral Ligament Knee Injury" OR "Knee Dislocation" OR "Meniscectomy" OR "Meniscus Injury" OR "Tibial Meniscus Injuries" OR "Meniscus Tear" OR "Bucket Handle Tear" OR "Flap Tear" OR "Patellar Tendinopathy" OR "Patellar Tendonitis" OR "Patellar Tendinosis" OR "Jumper Knee") AND ("Dry Needling" OR "Muscular Needling" OR "Intramuscular stimulation") NOT "Acupuncture"
Results: 38
**PEDro Search Formula**
Abstract & Title: Knee Pain, Patellofemoral Pain, Knee Osteoarthritis Therapy: Dry Needling Body part: Lower Leg or Knee Method: Clinical trial When Searching: AND
Results: 11
**SCOPUS Search Formula**
TITLE-ABS-KEY ("Patellofemoral Pain Syndrome" OR "Chondromalacia Patellae" OR "Knee Osteoarthritis" OR "Knee Arthritis" OR "Knee Arthroplasty" OR "Knee Prosthesis" OR "Knee Injury" OR "Anterior Cruciate Ligament Injury" OR "ACL Injury" OR "Medial Collateral Ligament Knee Injury" OR "Knee Joint Injury" OR "Meniscectomy" OR "Knee Dislocation" OR "Meniscus Injury" OR "Tibial Meniscus Injuries" OR "Meniscus Tear" OR "Bucket Handle Tear" OR "Flap Tear" OR "Patellar Tendinopathy" OR "Patellar Tendonitis" OR "Patellar Tendinosis" OR "Jumper Knee") AND TITLE-ABS-KEY ("Dry Needling" OR "Muscular needling" OR "Intramuscular stimulation")
Results: 52
**Cochrane Library Search Formula**
#1 "Patellofemoral Pain Syndrome"(MeSH Terms) #2 "Chondromalacia Patellae" #3 "Osteoarthritis, Knee"(MeSH Terms) #4 "Arthroplasty, Replacement, Knee"(MeSH Terms) #5 "Knee Arthroplasty" #6 "Knee Prosthesis"(MeSH Terms) #7 "Knee Injuries"(MeSH Terms) #8 "Anterior Cruciate Ligament Injuries"(MeSH Terms) #9 "ACL Injury" #10 "Medial Collateral Ligament Knee Injury" #11 "Knee Joint Injury" #12 "Knee Dislocation"(MeSH Terms) #13 "Meniscectomy"(MeSH Terms) #14 "Meniscus Injury" #15 "Tibial Meniscus Injuries"(MeSH Terms) #16 "Meniscus Tear" #17 "Bucket Handle Tear" #18 "Flap Tear" #19 "Patellar Tendinopathy" #20 "Patellar Tendonitis" #21 "Patellar Tendinosis" #22 "Jumper Knee" #23 "Dry Needling"(Mesh) #24 "Intramuscular stimulation" #25 "Muscular needling" #26 #1 OR #2 OR #3 OR #4 OR #5 OR #6 OR #7 OR #8 OR #9 OR #10 OR #11 OR #12 OR #13 OR #14 OR #15 OR #16 OR #17 OR #18 OR #19 OR #20 OR #21 OR #22 #27 #23 OR #24 OR #25 #28 #26 AND #27
Results: 42 Trials: 39

**Table 2:** Participant characteristics of included studies.

Study	Design	Group	Sample Size	Male/Female (%)	Age (years)	Pain Duration
**Patellofemoral Pain Syndrome**						
Abyaneh et al. 2016	RCT	G1 G2	17 17	NR NR	37.88 ± 9.53 33.58 ± 8	1.88 ± 1.16 years 2.11 ± 1.16 years
Espí-López et al 2017	RCT	G1 G2	30 30	15/15 16/14	29.7 ± 9.5 29.2 ± 10.5	9.5 ± 5.8 years 8.5 ± 6.3 years
Mason et al. 2017	RCT	G1 G2	20 19	20 17/2	20.3 ± 1.08 20.16 ± 2.12	17.75 ± 26.10 weeks 14.3 ± 16.36 weeks
Sutlive et al. 2018	RCT	G1 G2	30 30	56.7% male 66.7% male	30.3 ± 5.5 31.1 ± 5.1	27.4 ± 29.7 months 53.0 ± 66.8 months
Patel et al. 2019	RCT	G1 G2	35 35	NR NR	26 ± 5 33.3 ± 3	>3 months >3 months
Zarei et al. 2020	RCT	G1 G2	20 20	0/20 0/20	22.25 ± 3.25 25.65 ± 8.49	>3 months >3 months
**Knee Osteoarthritis**						
Itoh et al. 2008	RCT	G1 G2 G3	8 9 7	NR NR NR	74.2 ± 8.1 73.3 ± 6.5 70.5 ± 8.1	7.5 ± 6.0 years 6.1 ± 6.8 years 5.6 ± 5.1 years
Sánchez-Romero et al. 2019	RCT	G1 G2	31 31	21/10 23/8	72.97 ± 6.29 71.65 ± 5.00	62.88 ± 40.75 months 68.55 ± 30.31 months
**Post-Surgery Knee Pain**						
Mayoral et al. 2013	RCT	G1 G2	20 20	11/29	71.65 ± 6.06 72.90 ± 7.85	NR NR
Velázquez-Saornil et al. 2017	RCT	G1 G2	22 22	16/6 12/10	31.4 ± 8.3 34.4 ± 8.6	15.6 ± 1.5 days 15.5 ± 2.0 days

RCT: Randomized Controlled Trial; G1: Group 1; G2: Group 2; NR: Not reported.

**Table 3:** Description of the Dry Needling Intervention for Knee Pain Syndromes of the included studies.

Study	Group	TrP Criteria	Needle Approach	No. Punctures	Targeted Muscles	Gauge (mm)	Depth (mm)	Time	Frequency Incisions (Hz)	No. Incisions	LTR	Therapist
**Patellofemoral Pain Syndrome**												
Abyaneh et al. 2016	G1: Superficial Dry Needling (DN)	NO	At about 8 cm above the lateral femoral condyle of the knee joint line in vastus lateralis muscle At 8 cm above the medial femoral condyle of the knee joint line in vastus medialis muscle At 8 cm above the vase of the patella in the rectus femoris muscles.	3	Vastus lateralis Vastus medialis Rectus femoris	50 length	10	6	NR	NR	No	Physical therapist
Espí-López et al. 2017	G1: DN plus manual and exercise therapy	YES	Fast-in and fast-out technique at the active TrP	NR	Vastus lateralis Vastus medialis	0.32 × 40	15–20 vastus medialis 30–35 vastus lateralis	Until no more local twitch responses were elicited	1	NR	Yes	Physical therapist
Mason et al. 2017	G1: TrP DN	YES	Fast-in fast-out (Hong’s technique) at latent TrP	NR	Hamstrings’ muscles	NR	NR	NR	NR	NR	Yes	Physical therapist
	G2: TrP Sham DN	NO	At three points over the lateral hamstrings and three points over the medial hamstrings without the intention of locating any TrPs. The simulation was performed with a small nail	NA	Hamstrings’ muscles	NA	NA	NR	NA	NA	NA	Physical therapist
Sutlivee et al. 2018	G1: TrP DN	YES	Fast-in fast-out (Hong’s technique) at two TrP of each of three targeted quadriceps or the most painful location	6	Vastus medialis, rectus femoris and vastus lateralis	0.25 × 40	NR	5–10 s	NR	NR	Yes	Physical therapist
	G2: TrP Sham DN	YES	Simulation at the TrP without puncture		ipsilateral to the symptomatic knee	NA	NA	5–10 s	NA	NA	NA	Physical therapist
Patel et al. 2019	G1: TrP DN	YES	NR	NR	All trigger points of quadriceps muscle of the symptomatic knee	NR	NR	10 min	NR	NR	NR	Physical therapist
Zarei et al. 2020	G1: TrP DN	YES	Fast-in fast-out technique	NR	Gluteus medius Quadratus Lumborum	0.30 × 10 0.30 × 50	NR	NR	NR	NR	Yes	Physical therapist
**Knee Osteoarthritis**												
Itoh et al. 2008	G1: DN	YES	At the trigger points of the lumbar and lower extremity, using the “sparrow pecking” technique	3.3	Quadriceps, ilipsoas, sartorius, adductors, popliteus, gluteus minimus	0.2 × 50	10–30 mm	10 min	NR	NR	Yes	Acupuncturist
	G2: Sham DN	YES	At trigger points with steel needles, but the tips had been cut off to prevent the needle penetrating the skin. The acupuncturist inserted the needle and then used the sparrow pecking technique, then removed the needles	3.1	Quadriceps, ilipsoas, sartorius, adductors, popliteus, gluteus minimus	0.2 × 50	Not penetrating the skin	10 min	NR	NR	No	Acupuncturist
Sánchez-Romero et al. 2019	G1: DN plus exercise therapy	YES	At TrP, fast-in fast-out technique	NR	In all muscles with TrP of the symptomatic knees	0.30 × 40 0.30 × 60 0.30 × 75	According to the muscle selected and the subject	NR	NR	15	Yes	Physical Therapist
	G2: Sham DN plus exercise therapy	YES	Simulation	NR	In all muscles with TrP of the symptomatic knees	Sham Needle	NA	NA	NR	NA	NA	Physical Therapist
**Post-Surgery Knee Pain**												
Mayoral et al. 2013	G1: DN	YES	At TrP using Hong’s-fast-in fast-out technique	NR	Tensor fasciae latae, hip adductors, hamstrings, quadriceps gastrocnemius, popliteus	0.30 × 50	NR	NR	NR	20	Yes	Physical Therapist
	G2: Sham DN	NO	Simulated TrP DN	NA	NA	NA	NA	NA	NA	NA	NA	Physical Therapist
Velázquez-Saornil et al. 2017	G1: DN	YES	On the most active TrP of the vastus medialis of the affected knee, fast-in fast-out technique	1	Vastus Medialis	0.25 × 25	Varied according to the subject	1–2 min until LTR exhaustion, patient tolerance limit or 20 incisions	NR	20 incisions	Yes	Physical Therapy

**Table 4:** Score of randomized clinical trials with Physiotherapy Evidence Database (PEDro) scale.

	1	2	3	4	5	6	7	8	9	10	TOTAL
**Patellofemoral Pain Syndrome**											
Abyaneh et al. 2016	Y	N	Y	N	N	N	Y	Y	Y	N	5/10
Espí-López et al. 2017	Y	Y	Y	N	N	Y	Y	Y	Y	Y	8/10
Mason et al. 2017	Y	Y	Y	Y	N	Y	Y	Y	Y	Y	9/10
Sutlive et al. 2018	Y	Y	Y	Y	N	Y	Y	Y	Y	Y	9/10
Patel et al. 2019	Y	N	Y	N	N	N	Y	Y	Y	Y	6/10
Zarei et al. 2019	Y	Y	Y	N	N	Y	Y	Y	Y	Y	8/10
**Knee Osteoarthritis**											
Itoh et al. 2008	Y	N	Y	Y	N	Y	Y	N	Y	Y	7/10
Sánchez-Romero et al. 2019	Y	Y	Y	Y	N	Y	Y	N	Y	Y	8/10
**Post-Surgery Knee Pain**											
Mayoral et al. 2013	Y	N	Y	Y	N	Y	Y	Y	Y	Y	8/10
Velázquez-Saornil et al. 2017	Y	Y	Y	N	N	Y	Y	Y	Y	Y	8/10

1: Random Allocation of Participants; 2: Concealed Allocation; 3: Similarity Between Groups at Baseline; 4: Participant Blinding; 5: Therapist Blinding; 6: Assessor Blinding; 7: Fewer than 15% Dropouts; 8: Intention-to-Treat Analysis; 9: Between-Group Statistical Comparisons; 10: Point Measures and Variability Data. Y: Yes; N: No.

**Table 5:** Effects of dry needling on pain and related-disability for knee pain conditions.

Study	Intervention(s)	Sample Size	Intervention Duration (Sessions/Weeks)	Comparison and Outcome Measure	Between-Groups Differences (95%CI) (SMD)
**Patellofemoral Pain Syndrome**					
Abyaneh et al. 2016	G1: Superficial dry needling plus routine physical therapy G2: Routine physical therapy	N = 17 N = 17	DN: 5 ss 1 every two days for 10 days Routine Physical Therapy: 5 × 2 weeks	Pain (VAS) G1 vs. G2	0wk: −1.11 (−2.13, −0.09) (−0.72)
Espí-López et al. 2017	G1: Manual therapy and exercise plus dry needling G2: Manual therapy and exercise	N = 30 N = 30	1 × 3 weeks 1 × 3 weeks	Pain (NPRS) G1 vs. G2 G1 vs. G2 Pain (KOOS) G1 vs. G2 G1 vs. G2 Symptoms (KOOS) G1 vs. G2 G1 vs. G2 Function in daily living (KOOS) G1 vs. G2 G1 vs. G2 Function in sport and recreation (KOOS) G1 vs. G2 G1 vs. G2 Quality of life (KOOS) G1 vs. G2 G1 vs. G2 Disability (IKDC) G1 vs. G2 G1 vs. G2 Pain (IKDC pain subscale) G1 vs. G2 G1 vs. G2 Function (IKDC function subscale) G1 vs. G2 G1 vs. G2	15d: −0.3 (−0.9, 0.3) (−0.09) 3mo: 0.3 (−0.2, 0.8) (0.09) 15d: −2.9 (−5.8, 0.0) (−0.) 3mo: −2.1 (−4.6, 0.4) (−0.13) 15d: −0.7 (−2.4, 1.0) (−0.06) 3mo: −0.8 (−1.9, 0.3) (−0.06) 15d: −0.9 (−1.8, 0.0) (−0.08) 3mo: −2.8 (−5.7, 0.1) (−0.21) 15d: 0.2 (−1.0, 1.4) (0.01) 3mo: −3.2 (−6.4, 0.0) (−0.16) 15d: 1.2 (−1.0, 3.4) (0.14) 3mo: 3.5 (−0.5, 7.5) (0.21) 15d: 2.9 (0.0, 5.8) (0.19) 3mo: 2.3 (−0.1, 4.7) (0.17) 15d: 1.9 (−2.0, 5.8) (0.18) 3mo: 0.2 (−0.1, 0.5) (0.02) 15d: −2.3 (−6.0, 1.4) (−0.24) 3mo: 1.5 (0.0, 3.0) (0.36)
Mason et al. 2017	G1: Dry needling and Stretching G2: Sham Dry Needling and Stretching	N = 20 N = 19	2 × 1 week 2 × 1 week	Deep squat pain (VAS) G1 vs. G2 Step down pain (VAS) G1 vs. G2 Disability (LEFS) G1 vs. G2 Active Knee Extension G1 vs. G2 Active Straight Leg Raise G1 vs. G2 Deep squat range of motion G1 vs. G2	7d: −6.00 (−17.80, 5.80) (−0.31) 7d: −6.80 (−16.63, 3.03) (−0.44) 7d: 3.04 (−2.70, 8.78) (0.33) 7d: 0.31 (−6.23, 6.85) (0.03) 7d: 0.04 (−5.12, 5.20) (0.00) 7d: 3.38 (−10.50, 17.26) (0.15)
Sutlive et al. 2018	G1: DN and isometric and stretching quadriceps home-exercises G2: Sham DN and isometric and stretching quadriceps home-exercises	N = 30 N = 30	1 session 1 session	Pain squat (NPRS) G1 vs. G2 Pain upstairs (NPRS) G1 vs. G2 Pain down stairs (NPRS) G1 vs. G2 Disability function (LEFS) G1 vs. G2 Disability (Kujala) G1 vs. G2	72hr: 0.60 (−0.40, 1.60) (0.30) 72hr: 0.00 (−0.72, 0.72) (0.00) 72hr: 0.40 (−0.24, 1.04) (0.31) 72hr: 3.50 (−2.90, 9.90) (0.28) 72hr: 6.20(0.21, 12.19) (0.52)
Patel et al. 2019	G1: Dry needling G2: Ultrasound	N = 35 N = 35	DN: 1 session Ultrasound: 1 session	Pain (NPRS) G1 vs. G2 Sensitivity (PPT) G1 vs. G2	0wk: −0.97 (−1.60, −0.34) (−0.71) 0wk: 5.28 (2.57, 7.99) (0.90)
Zarei et al. 2019	G1: Dry needling plus exercise program G2: Exercise program	N = 20 N = 20	DN: 1 × 4 weeks Exercise program: 5 × 4 weeks	Pain (NPRS) G1 vs. G2 G1 vs. G2 Disability (Kujala) G1 vs. G2 G1 vs. G2 Pain sensitivity (PPT gluteus medium) G1 vs. G2 G1 vs. G2 Pain sensitivity (PPT quadratus lumborum) G1 vs. G2 G1 vs. G2 Step-down test G1 vs. G2 G1 vs. G2 SEBT anterior G1 vs. G2 G1 vs. G2 SEBT posterolateral G1 vs. G2 G1 vs. G2 SEBT posteromedial G1 vs. G2 G1 vs. G2	0wk: −2.00 (−2.63, −1.37) (−1.94) 2wk: −2.10 (−2.68, −1.52) (−2.18) 0wk: 8.00 (4.51, 11.49) (1.39) 2wk: 11.35 (8.07, 14.63) (2.10) 0wk: 2.97 (2.53, 3.41) (4.09) 2wk: 3.45 (3.08, 3.82) (5.68) 0wk: 2.75 (2.29, 3.21) (3.64) 2wk: 2.88 (2.53, 3.23) (4.93) 0wk: 6.45 (4.03, 8.87) (1.62) 2wk: 7.15 (5.18, 9.12) (2.20) 0wk: 0.08 (0.00, 0.16) (0.62) 2wk: 0.09 (0.02, 0.16) (0.73) 0wk: 0.05 (−0.02, 0.12) (0.43) 2wk: 0.08 (0.01, 0.15) (0.71) 0wk: 0.08 (0.02, 0.14) (0.78) 2wk: 0.08 (0.02, 0.14) (0.78)
**Knee Osteoarthritis**					
Itoh et al. 2008	G1: Trigger Point Dry needling G2: AcupunctureG3: Sham Dry Needling	N = 8 N = 9 N = 7	1 × 5 weeks 1 × 5 weeks 1 × 5 weeks	Pain (VAS) G1 vs. G2 G1 vs. G2 G1 vs. G2 G1 vs. G3 G1 vs. G3 G1 vs. G3 G2 vs. G3 G2 vs. G3 G2 vs. G3 Disability (WOMAC) G1 vs. G2 G1 vs. G2 G1 vs. G2 G1 vs. G3 G1 vs. G3 G1 vs. G3 G2 vs. G3 G2 vs. G3 G2 vs. G3	5wk: −1.65 (−3.28, −0.02) (−0.91) 10wk: −0.50 (−2.25, 1.25) (−0.25) 20wk: −0.10 (−1.17, 0.97) (−0.08) 5wk: −3.10 (−4.48, −1.72) (−2.09) 10wk: −1.25 (−3.14, 0.64) (−0.65) 20wk: −0.90 (−2.61, 0.81) (−0.52) 5wk: −1.45 (−2.88, −0.02) (−0.89) 10wk: −0.75 (−2.40, 0.90) (−0.39) 20wk: −0.80 (−2.51, 0.91) (−0.47) 5wk: −12.31 (−21.98, −2.63) (−1.19) 10wk: −5.90 (−16.06, 4.26) (−0.53) 20wk: −1.00 (−8.39, 6.39) (−0.12) 5wk: −18.45 (−28.92, −7.98) (−1.63) 10wk: −9.70 (−20.61, 1.21) (−0.84) 20wk: −4.00 (−14.87, 6.87) (−0.37) 5wk: −6.14 (−13.37, 1.09) (−0.81) 10wk: −3.80 (−13.43, 5.83) (−0.37) 20wk: −3.00 (−15.04, 9.04) (−0.24)
Sánchez-Romero et al. 2019	G1: Trigger Point Dry needling plus therapeutic exercise G2: Sham Dry Needling plus therapeutic exercise	N = 31 N = 31	1 × 6 weeks Therapeutic exercise: 24 sessions for 12 weeks 1 × 6 weeks Therapeutic exercise: 24 sessions for 12 weeks	Pain (NPRS) G1 vs. G2 G1 vs. G2 G1 vs. G2 G1 vs. G2 G1 vs. G2 Disability (WOMAC) G1 vs. G2 G1 vs. G2 G1 vs. G2 G1 vs. G2 G1 vs. G2 Quality of life (EQ-5D) G1 vs. G2 G1 vs. G2 G1 vs. G2 G1 vs. G2 G1 vs. G2 Barthel Index G1 vs. G2 G1 vs. G2 G1 vs. G2 G1 vs. G2 G1 vs. G2 Time Up and Go Test G1 vs. G2 G1 vs. G2 G1 vs. G2 G1 vs. G2 G1 vs. G2 Medication consumption G1 vs. G2	0wk: −0.17 (−1.33, 0.99) (−0.07) 3mo: −0.65 (−2.22, 0.92) (−0.20) 6mo: −0.80 (−2.45, 0.85) (−0.24) 9mo: −0.32 (−1.67, −1.0.3) (−0.12) 12mo: −0.20 (−1.02, 0.26) (−0.06) 0wk: −3.39 (−10.56, 3.78) (−0.23) 3mo: −4.36 (−11.25, 2.53) (−0.31) 6mo: −4.23 (−12.07, 3.61) (−0.27) 9mo: −4.06 (−11.55, 3.43) (−0.27) 12mo: −3.32 (−10.77, 4.13) (−0.22) 0wk: −0.77 (−2.05, 0.51) (−0.30) 3mo: −0.60 (−2.02, 0.82) (−0.21) 6mo: −0.70 (−1.97, 0.57) (−0.27) 9mo: −0.73 (−1.90, 0.44) (−0.31) 12mo: −0.50 (−1.95, 0.95) (−0.17) 0wk: 0.58 (−1.74, 2.90) (0.12) 3mo: 0.96 (−2.05, 3.97) (0.16) 6mo: 0.09 (−2.20, 2.38) (0.02) 9mo: 0.07 (−1.76, 1.90) (0.02) 12mo: −0.06 (2.27,.15) (−0.01) 0wk: −0.22 (−1.42, 0.98) (−0.09) 3mo: −0.23 (−1.61, 1.15) (−0.08) 6mo: −0.16 (−152, 1.20) (−0.06) 9mo: −0.58 (−1.83, 0.67) (−0.23) 12mo: −0.45 (−1.77, 0.87) (−0.17) 12mo: −1.62 (−2.79, −0.45) (−0.68)
**Post-Surgery Knee Pain**					
Mayoral et al. 2013	G1: Trigger Point Dry needling G2: Sham Dry Needling	N = 22 N = 22	1 session 1 session	Pain (VAS) G1 vs. G2 G1 vs. G2 G1 vs. G2 Pain (WOMAC) G1 vs. G2 G1 vs. G2 G1 vs. G2 Stiffness (WOMAC) G1 vs. G2 G1 vs. G2 G1 vs. G2 Disability (WOMAC) G1 vs. G2 G1 vs. G2 G1 vs. G2 ROM G1 vs. G2 Strength (Flexion) G1 vs. G2 Strength (Extension) G1 vs. G2	1mo: −0.85 (−2.42, 0.72) (−0.33) 3mo: −0.47 (−1.76, 0.82) (−0.22) 6mo: 0.27 (−1.01, 1.54) (0.13) 1mo: 0.93 (−1.21, 3.07) (0.26) 3mo: 1.24 (−0.54, 3.02) (0.42) 6mo: 0.11 (−1.67, 1.89) (0.04) 1mo: 0.09 (−0.81, 0.99) (0.06) 3mo: 0.07 (−1.01, 1.15) (0.04) 6mo: 0.09 (−1.17, 0.97) (−0.08) 1mo: 4.02 (−1.91, 9.95) (0.41) 3mo: 3.18 (−3.61, 9.97) (0.28) 6mo: −0.83 (−6.82, 5.16) (−0.08) 1mo: −3.01 (−13.87, 7.85) (−0.17) 1mo: −0.76 (−4.62, 3.10) (−0.12) 1mo: −1.10 (−5.27, 3.07) (−0.16)
Velázquez-Saornil et al. 2017	G1: Rehabilitation plus TrP DN G2; Rehabilitation alone	N = 22 N = 22	DN: 1 session Rehabilitation: 5 × 5 weeks	Pain (VAS) G1 vs. G2 Disability (WOMAC) G1 vs. G2 ROM G1 vs. G2 Balance (SEBT) G1 vs. G2	0wk: −0.48 (−1.08, 0.12) (−0.47) 0wk: −4.52 (−6.76, −2.28) (−1.17) 0wk: 2.86 (0.03, 5.69) (0.60) 0wk: 2.30 (−0.79, 5.39) (0.44)

G: Group; PSFS: Patient-Specific Functional Scale; LEFS: Lower Extremity Functional Scale; VAS: Visual analogue scale; PPT: Pressure Pain threshold; SEBT: Star Excursion Balance Test; ROM: Range of Motion; WOMAC: Western Ontario and McMaster Universities Osteoarthritis Index; SDT: Step-down test; EQ-5D: EuroQol 5-Dimension Self-Report Questionnaire; NR: Not reported; wk: weeks; Mo: months; hr: hours; d: days; ^±^ Values estimated from graphs.

**Table 6:** Grading of Recommendations Assessment, Development, and Evaluation (GRADE) Evidence profile for the effects of dry needling for knee pain conditions.

Number of Studies	Risk of Bias	Inconsistency	Indirectness of Evidence	Imprecision	Publication Bias	Quality of Evidence	SMD (95% CI)
**Effects of Dry Needling on Knee Pain at Short-term**							
**Overall effect** (n = 10)	No	Serious (I^2^ = 68%)	No	No	No	Moderate	−0.53 (−0.87, −0.19) *
Patellofemoral Pain (n = 6)	No	Serious (I^2^ = 80%)	No	No	No	Moderate	−0 64 (−1.17, −0.11) *
Knee Osteoarthritis (n = 2)	No	Serious (I^2^ = 52%)	No	Very serious	No	Very Low	−0.37 (−1.15, 0.41)
Post-Surgery Knee Pain (n = 2)	No	No (I^2^ = 0%)	No	Very serious	No	Low	−0.40 (−0.84, 0.04)
**Effects of Dry Needling on Knee Pain at Mid-term**							
**Overall effect** (n = 4)	No	No (I^2^ = 0%)	No	Very Serious	No	Low	−0.11 (−0.41, 0.18)
Patellofemoral Pain (n = 1)	No	No	No	Very Serious	No	Low	0.09 (−0.42, 0.60)
Knee Osteoarthritis (n = 2)	No	No (I^2^ = 0%)	No	Very Serious	No	Low	−0.21 (−0.66, 0.23)
Post-Surgery Knee Pain (n = 1)	No	No	No	Very Serious	No	Low	−0.22 (−0.84, 0.40)
**Effects of Dry Needling on Knee Pain at Long-term**							
**Overall effect** (n = 3)	No	No (I^2^ = 0%)	No	Very Serious	No	Low	−0.00 (−0.36, 0.36)
Knee Osteoarthritis (n = 2)	No	No (I^2^ = 0%)	No	Very Serious	No	Low	−0.07 (−0.51, 0.37)
Post-Surgery Knee Pain (n = 1)	No	No	No	Very Serious	No	Low	0.13 (−0.49, 0.75)
**Effects of Dry Needling on Related** **Disability at Short-term**							
**Overall effect** (n = 8)	No	Serious (I^2^ = 80%)	No	No	No	Moderate	−0.58 (−1.08, −0.09) *
Patellofemoral Pain (n = 4)	No	Very Serious (I^2^ = 85%)	No	No	No	Low	−0 69 (−1.46, 0.09)
Knee Osteoarthritis (n = 2)	No	Serious (I^2^ = 61%)	No	Very serious	No	Very Low	−0.60 (−1.51, 0.32)
Post-Surgery Knee Pain (n = 2)	No	Very Serious (I^2^ = 91%)	No	Very serious	No	Very Low	−0.37 (−1.92, 1.17)
**Effects of Dry Needling on Related** **Disability at Mid-term**							
**Overall effect** (n = 4)	No	No (I^2^ = 0%)	No	Very Serious	No	Low	−0.10 (−0.39, 020)
Patellofemoral Pain (n = 1)	No	No	No	Very Serious	No	Low	−0.01 (−0.52, 0.49)
Knee Osteoarthritis (n = 2)	No	No (I^2^ = 0%)	No	Very Serious	No	Low	−0.36 (−0.80, 0.09)
Post-Surgery Knee Pain (n = 1)	No	No	No	Very Serious	No	Low	0.28 (−0.34, 0.91)
**Effects of Dry Needling on Related** **Disability at Long-term**							
**Overall effect** (n = 3)	No	No (I^2^ = 0%)	No	Very Serious	No	Low	−0.16 (−0.52, 0.20)
Knee Osteoarthritis (n = 2)	No	No (I^2^ = 0%)	No	Very Serious	No	Low	−0.20 (−0.64, 0.25)
Post-Surgery Knee Pain (n = 1)	No	No	No	Very Serious	No	Low	−0.08 (0.70, 0.54)
